# AnchoisFert: A New Organic Fertilizer from Fish Processing Waste for Sustainable Agriculture

**DOI:** 10.1002/gch2.202100141

**Published:** 2022-03-02

**Authors:** Adele Muscolo, Francesco Mauriello, Federica Marra, Paolo Salvatore Calabrò, Mariateresa Russo, Rosaria Ciriminna, Mario Pagliaro

**Affiliations:** ^1^ Dipartimento di Agraria Università Mediterranea di Reggio Calabria Feo di Vito Reggio Calabria 89124 Italy; ^2^ Dipartimento di Ingegneria Civile Energia Ambiente e Materiali Università Mediterranea di Reggio Calabria via Graziella, Feo di Vito Reggio Calabria 89122 Italy; ^3^ Istituto per lo Studio dei Materiali Nanostrutturati CNR via U. La Malfa 153 Palermo 90146 Italy

**Keywords:** AnchoisFert, anchovy, circular economy, fish waste, organic fertilizers

## Abstract

The “AnchoisFert”, the solid residue comprised of milled anchovy leftovers after fish oil extraction with biobased limonene, is a powerful organic fertilizer. Employed to promote the growth of Tropea's red onion (*Allium cepa*), the fertilizer turns out to largely be superior to commonly used organic (manure) and chemical (nitrogen phosphorous potassium) fertilizers. Rich in proteins, organic carbon, flavonoids, magnesium, potassium, phosphate and sulfate, and devoid of antibiotics and antibiotic resistance genes, the new organic fertilizer can replace both conventional organic and inorganic fertilizers. This discovery closes the fishing material cycle for the most fished species across the seas opening the route to a new class of organic fertilizers of exceptional performance derived from abundant biowaste via a low‐cost and environmentally‐friendly circular economy process.

## Graphical abstract

The “AnchoisFert”, the solid residue comprised of milled anchovy leftovers after fish oil extraction with biobased limonene, is shown to be a powerful organic fertilizer, rich in proteins, organic carbon, flavonoids, magnesium, potassium, phosphate, and sulfate. This discovery closes the fishing material cycle for the most fished species across the seas.

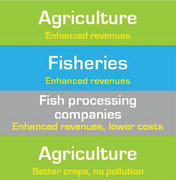

## 1 Introduction

Rich in essential proteins and essential polyunsaturated (“omega‐3”) lipids, vitamin D, iodine, iron, calcium, zinc and other micronutrients, fish, shrimp and other marine species are fundamental components of human diets across virtually all world's countries.^[^ ^1^ ^]^ Between 1961 and 2017 the world's population went from less than 3.1 billion to 7.6 billion people. In the same period, per capita food fish consumption rose from 9.0 (live weight equivalent) to 20.3 kg.^[^ ^2^ ^]^ Not surprisingly, in 2018 the global fish production reached the staggering 179 million tonne threshold, 156 million of which used for human consumption.^[^ ^2^ ^]^ This remarkable growth was made possible also by aquaculture which expanded fish availability to regions and countries with limited access to the cultured species.

Unfortunately, the aforementioned growth in seafood demand has been met also by the growth of overfishing. Recently, the share of stocks overfished was found to vary from 59% in the Mediterranean and Black Sea, to >40% in the southwest, eastern and western central Atlantic, and southeast Pacific oceans.^[^ ^3^ ^]^ Besides fish populations and fisheries, overfishing affects marine ecosystems functioning in general.^[^ ^4^ ^]^

Efforts aimed at improving the overall (economic, social, and environmental) sustainability of fishing include the economic valorization of processing waste. About 25% of the global amount of the total fish capture every year is not suitable for consumption (bones, shell, intestine, fin, heads, tails, and skin).^[^ ^5^ ^]^ This fish processing waste is chiefly used as raw material for the production of fish oil and of fishmeal. However, only 25% of fish waste is currently converted into fishmeal products that has been estimated to modestly increase from 22% to 28% (and that of fish oil from 40% to 45%).^[^ ^2^ ^]^

Selected fish processing companies (e.g., tuna processing companies) sell fish biowaste to pet food producers. Most fish processing companies, however, face negative costs to get rid of their processing waste. In Italy, for example, in 2020 the typical tariff paid by a fish processing company for biowaste disposal was €0.2/kg.^[^ ^6^ ^]^

Plentiful research has been devoted in the last two decades to develop methods to convert these wastes into high value products such as fish oil, fish hydrolysate, collagen, chitin, chitosan, and hydroxyapatite.^[^ ^7^ ^]^ In this context, a circular economy process for the extraction of fish oil from anchovy leftovers using biobased limonene as sole extraction solvent was introduced in Sicily in 2019.^[^ ^8^ ^]^ After extraction, the agrosolvent derived from the citrus fruit processing industry is nearly entirely recovered, whereas the new oil rich of polyunsaturated fatty acids and oleic acid in their natural triglyceride form, and vitamin D in vitamin D_3_ most bioavailable form,^[^ ^9^ ^]^ is ready for producing new dietary supplements and likely new therapeutic treatments (see below).

Though generally using highly refined lipids in diethyl ester form as active ingredients,^[^ ^10^ ^]^ omega‐3 lipids are widely consumed worldwide as dietary supplements due the unbalanced diet in most of the world's countries which has created what Winkler has called “the most hidden hunger”,^[^ ^11^ ^]^ namely that of docosahexaenoic acid (DHA) and eicosapentaenoic acid (EPA) present in fish oils in triglyceride form.

Anchovies are the most fished species in the world. Stocks are threatened in many countries. For example, in Spain's and France's Bay of Biscay the anchovy fishing was forbidden between July 2005 and early 2010 when it reopened with a total allowable catch of only 7000 tonnes. In 2019, however, the catch jumped to nearly 27000 metric tons, with the biomass of all spawning‐age anchovies in 2020 being the highest since the Biscay anchovy surveys began in 1987.^[^ ^12^ ^]^ The recovery of the anchovy population was caused by the closure of the fishery for five years.^[^ ^13^ ^]^ Anchovies fished in the Biscay Bay or in the Mediterranean sea are used to produce renowned anchovy fillets. About 50% of the whole fish is converted into fillet. The leftovers are disposed of as biowaste at high economic costs. Clearly, providing fisheries with extra revenues originating from the valorisation of anchovy fillet leftovers would reduce the risk of overfishing, especially when producing high added value bioproducts from said biowaste.

**Scheme 1:**
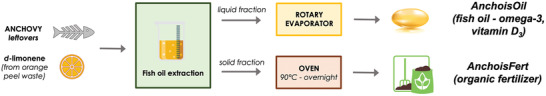
Overall upgrading process of anchovy fillet leftovers converted into AnchoisOil and AnchoisFert.

One such product is the new “AnchoisOil” fish oil extracted from anchovy leftovers with biobased *d*‐limonene only (**Scheme** **1**).

Rich in oleic acid, DHA, and EPA in natural triglyceride form, the oil contains also plentiful vitamin D in its most bioavailable form (vitamin D_3_), and a carotenoid imparting the orange color whose nature and amount will be shortly reported. Encapsulated in silica, the new oil acts as a promising antiproliferative agent against lung cancer cells.^[^ ^14^ ^]^

The oily fraction extracted with limonene, however, comprises only 10 weight percent of the anchovy leftover. Aware of the rich protein, mineral, and carbon content of the extraction residue, we therefore investigated its employment as organic fertilizer.

Generally commercialized in the form of nitrates, phosphates, and potassium salt powder mixtures, chemical fertilizers are widely used across the world to maximize crop yields. Their use, however, poses significant environmental and health problems due to accumulation of heavy metals, inorganic acids, and organic pollutants in soil^[^ ^15^ ^]^ and groundwater,^[^ ^16^ ^]^ and depletion of organic carbon in agricultural soils.

Furthermore, the cost of chemical fertilizers is high (and continuously growing) making their use economically not viable for many farmers. In brief, the replacement of chemical nutrients with organic fertilizers is highly desirable. The main problem with the latter fertilizers, however, is the poor performance as their effect on crop yield is often slow and variable.^[^ ^17^ ^]^ Highly effective and economically viable organic fertilizers would be in high demand. Along with a strengthened membership of farmer organizations and better policy measurements,^[^ ^18^ ^]^ their development might convince farmers to replace chemical with organic fertilization.

In the following we report the discovery that the solid residue of anchovy fillet leftovers extracted with biobased limonene, named herein “AnchoisFert”, is a powerful fertilizer capable of promoting the growth of Tropea's red onion (*Allium cepa*) significantly better than commonly used chemical and organic fertilizers. The discovery closes the fishing material cycle for anchovy fishing and dramatically increases the sustainability of both anchovy fishing and agriculture.

## 2 Results and Discussion

Anchovy leftovers obtained after fish oil extraction with limonene, nitrogen phosphorous potassium (NPK) and horse manure (HM) were chemically and physically characterized with respect to the main parameters of relevance to agriculture, namely pH, electrical conductivity, main ions, carbon, nitrogen, total flavonoid, and phenol content. Data in **Table** **1** are expressed in units per gram dried weight (DW). The residue of the limonene extraction is mildly acidic (pH is 6.27) and has poor electrical conductivity (5.9 µS cm^−1^). The residue has a very high content of both carbon (40%) and nitrogen (12%) that, along with the large amount of valued mineral nutrients, in particular calcium (35.2 mg g^−1^) and sulfate (16.2 mg g^−1^), but also magnesium (6.7 mg g^−1^), potassium (5.5 mg g^−1^), and phosphate (5.6 mg g^−1^), is very promising in light of its employment as fertilizer. Additionally, it contains also bioactive compounds such as total phenols (8507 µg g^−1^ total phenol content expressed as tannic acid, TA, per gram DW) and total flavonoids (1868 µg g^−1^ total flavonoid content [TFC] expressed as quercetin equivalent, QE, per gram DW), with well‐known antioxidant activity.

**Table 1:** Main chemical and physical parameters of anchovy leftovers after oil extraction with limonene

	AF	NPK	HM
Chemical parameter	Value	Value	Value
Total solids (TS)	20.1%	–	–
Volatile solids (VS)	66.7%	–	–
C	40%	–	30%
N	12%	10%	2%
C/N	3.3	–	15%
Total phenols	8507 µg TA/g DW	–	637.5
Total flavonoids	1868 µg QE/mg		549.5
pH	6.27		7.32
Electrical conductivity	5.945 µS cm^−1^		6.834 µS cm^−1^
Ca^2+^	35.2 mg g^−1^		0.4
K^+^	5.5 mg g^−1^	100 mg g^−1^	2.2 mg g^−1^
Mg^2+^	6.7 mg g^−1^	–	0.12
SO_4_^2−^	16.2 mg g^−1^	–	0.6
PO_4_^3−^	5.6 mg g^−1^	100 mg g^−1^	3.1 mg g^−1^
Limonene	0.140 mg g^−1^	–	–

**Table** **2** lists the chemical parameters of non‐fertilized soil (CTR), chemically‐fertilized (NPK), and organically‐fertilized soils with HM and AnchoisFert (AF), analyzed after onion harvesting (3 months). Data are expressed in units per gram of dried soil (DS).

**Table 2:** Chemical and biochemical parameters of non‐fertilized (CTR), chemically‐fertilized (NPK), and organically‐fertilized (HM and AF) soils after onion harvesting

Fertilizer	pH (H_2_O)	pH (KCl)	EC [µS cm^−1^]	TPC [µg TA g^−1^ DS]	DHA [µg TPF g^−1^ DS h^−1^]	FDA [µg fluorescein g^−1^ DS]	C [%]	N [%]	OM [%]	C/N
CTR	8.06^a^ ^)^	6.98^a^ ^)^	436^a^ ^)^	351^c)^	1.66^b)^	17.6^c)^	2.52^c)^	0.22^b)^	4.33^c)^	11^c)^
NPK	8.14^a^ ^)^	6.99^a^ ^)^	438^a^ ^)^	407^b)^	2.08^a^ ^)^	21.8^b)^	3.21^b)^	0.20^b)^	5.52^b)^	16^a^ ^)^
HM	7.97^a^ ^)^	6.98^a^ ^)^	391^b)^	380^b)^	1.68^b)^	16.9^c)^	3.54^a^ ^)^	0.23^b)^	6.08^a^ ^)^	15^a,b)^
AF	8.19^a^ ^)^	6.89^a^ ^)^	357^c)^	550^a^ ^)^	1.48^c)^	32.6^a^ ^)^	3.59^a^ ^)^	0.26^a^ ^)^	6.19^a^ ^)^	14^b)^

^a)^ Data expressed as mean of three replicates. Different letters indicate significant differences at *p* < 0.05.

The anchovy‐based fertilizer limited the increase of the electrical conductivity (357 µS cm^−1^) probably as a result of the residual limonene present in the leftovers after the omega‐3 extraction. The soil treated with it had the highest total phenol (550 µg TAE g^−1^DS), carbon (3.59%), and nitrogen (0.26%) content. Likely due to specific inhibition of dehydrogenase enzyme by phenols contained in AnchoisFert, as it happens with shikimate dehydrogenase inhibited by several polyphenols,^[^ ^19^ ^]^ the dehydrogenase activity (DHA) of soil fertilized with AF was the lowest (1.48 µg TPF g^−1^ DS h^−1^) among all studied soils. Indeed, the fact that most phenolics and flavonoids contained in AnchoisFert are beneficial to soil microbial activity was clearly shown by total microbial activity measured indirectly by using fluorescein diacetate (FDA) hydrolysis which was the highest (32.6 µg fluorescein g^−1^DS) for the soil fertilized with AF.

For comparison, the soil fertilized with NPK and HM respectively contained 21.8 and 16.9 µg fluorescein diacetate g^−1^ DS. Furthermore, soil organic matter (OM) reached the highest value (6.19%) in soil fertilized with AnchoisFert. We briefly remind that OM is an indicator of soil quality directly linked to the soil's ability to work as nutrient sink and promote biological activity affecting the supply of energy for microbial growth and enzyme production.^[^ ^20^ ^]^

The analysis of cations and anions in non‐fertilized (CTR), chemically‐fertilized (NPK), and organically‐fertilized (HM and AF) soils analyzed after onion harvesting (3 months) presented significant differences (**Table** **3**, data expressed in units per gram of DS).

**Table 3:** Cations and anions in non‐fertilized (CTR), chemically‐fertilized (NPK), and organically‐fertilized (HM and AF) soils after onion harvesting

Cation	CTR (mg g^−1^)	NPK (mg g^−1^)	HM (mg g^−1^)	AF (mg g^−1^)
NH_4_^+^	0.0045 ± 0.02^a^ ^)^	0.059 ± 0.01^a^ ^)^	0.061 ± 0.01^a^ ^)^	0.072 ± 0.02^a^ ^)^
K^+^	1.4 ± 0.1^b)^	1.8 ± 0.2^a^ ^)^	1.9 ± 0.3^a^ ^)^	1.9 ± 0.2^a^ ^)^
Mg^2+^	0.11 ± 0.07^c)^	0.22 ± 0.06^b)^	0.21 ± 0.05^b)^	0.33 ± 0.04^a^ ^)^
Ca^2+^	0.29 ± 0.09^a^ ^)^	0.21 ± 0.1^a^ ^)^	0.34 ± 0.1^a^ ^)^	0.44 ± 0.7^a^ ^)^
Anion	CTR [mg g^−1^]	NPK [mg g^−1^]	HM [mg g^−1^]	AF [mg g^−1^]
F^−^	0.01 ± 0.01^a^ ^)^	0.03 ± 0.01^a^ ^)^	0.006 ± 0.0^b)^	0.01 ± 0.01^a^ ^)^
Cl^−^	0.14 ± 0.01^d)^	0.4 ± 0.07^c)^	0.7 ± 0.01^b)^	1.2 ± 0.1^a^ ^)^
Br^−^	Nd	0.03^a^ ^)^	0.03^a^ ^)^	0.03^a^ ^)^
NO_3_^−^	0.09 ± 0.01^b)^	0.3 ± 0.02^a^ ^)^	0.5 ± 0.02^a^ ^)^	0.4 ± 0.02^a^ ^)^
NO_2_^−^	0.07 ± 0.01^a,c)^	0.07 ± 0.02^a^ ^)^	0.07 ± 0.01^a^ ^)^	0.07 ± 0.01^a^ ^)^
SO_4_^2−^	0.1 ± 0.01^c)^	3.5 ± 1.2^a,b)^	2 ± 0.3^b)^	4.1 ± 2^a^ ^)^

^a)^ Data expressed as mean of three replicates. Different letters indicate significant differences at *p* < 0.05.

In general, treatment with AF resulted in the largest increases with respect to all cations measured. A 16‐fold increase (from 0.45 × 10^−2^ to 7.2 × 10^−2 ^mg g^−1^) was measured for ammonium in soils fertilized with AnchoisFert, followed by a 13.5‐fold increase in the presence of horse manure, which is rich in urea and ammonia. A 35% increase in potassium was observed in soils treated with both organic fertilizers (AF and HM). This increase was higher (35% versus 28%) than that measured in the presence of the NPK chemical fertilizer containing nearly pure potassium nitrate. The threefold increase in magnesium in soil fertilized with the new fertilizer was particularly significant, and higher than the twofold increase in the presence of the NPK fertilizer. Finally, a 1.5‐fold increase in calcium was observed for soil fertilized with AF, followed by a 1.17 increase for the soil fertilized with HM.

Getting to anions, it is important to notice that the amount of fluoride remained unvaried to a very low level (0.01 mg g^−1^) for bulbs grown in non‐treated soil and soil fertilized with AF, whereas it even decreased by 40% when going from the control soil to soil fertilized with horse manure (from 1 × 10^−2^ to 0.6 × 10^−2 ^mg g^−1^). Similar good results were measured for nitrite whose content remained unvaried to 7 × 10^−2 ^mg g^−1^ in all bulbs, independent of fertilization with chemical or organic fertilizers.

A significant increase in bulbs from plants grown in organically fertilized soils was observed for both chloride and nitrate ions. In detail, chloride went from 0.14 mg g^−1^ to 1.2 and 0.7 mg g^−1^ upon treatment with AF and HM, respectively. The 5.5‐fold increase in nitrate (from 0.09 to 0.5 mg g^−1^) was the largest for bulbs grown in plants in soils treated with HM, followed by the 4.4‐fold increase for bulbs grown in plants in the presence of AF.

These increases, once again, were larger than the 3.3‐fold increase measured in bulbs from plants grown in soil treated with the NPK fertilizer rich in concentrated nitrate ions. Finally, a remarkable 41‐fold increase in sulfate ion was observed for bulbs grown in soil fertilized with AnchoisFert, followed by a 35‐fold increase for bulbs from soil fertilized with the chemical fertilizer NPK containing abundant sulfate anions.

The plant growth parameters of red onion grown in pots for 3 months (maturation time) with chemical fertilizer NPK (20:10:10; 1.2 g/pot), horse manure (13 g/pot), and AnchoisFert (1.60 g/pot) showed significant differences (**Table** **4**).

**Table 4:** Growth parameters of red onion grown for 3 months in non‐fertilized (CTR), chemically (NPK), horse manure (HM), and AnchoisFert (AF) fertilizer

Parameter	CTR	NPK	HM	AF
Bulb weight [g]	82 ± 2^c)^	102 ± 5^c)^	101 ± 4^b)^	136 ± 5^a^ ^)^
Bulb diameter [cm]	3 ± 1^b)^	4 ± 1^b)^	4 ± 0.7^b)^	7 ± 1^a^ ^)^
Leaf length [cm]	42 ± 3^a^ ^)^	46 ± 2^a^ ^)^	42 ± 2^a^ ^)^	46 ± 2^a^ ^)^
Average plant height [cm]	55 ± 2^b)^	64 ± 3^a^ ^)^	63 ± 3^a^ ^)^	68 ± 2^a^ ^)^

^a)^ Data expressed as mean ± standard error. Different letters indicate significant differences at *p* < 0.05.

**Figure 1:**
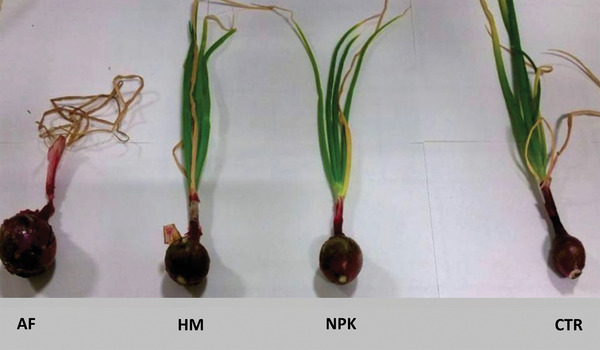
Tropea's red onion plant grown in soil fertilized with AnchoisFert (left), horse manure (HM), nitrogen:phosphorous:potassium (NPK), and in unfertilized soil (CTRL).

With the exception of the leaf length that remained unvaried to 46 cm, once again fertilization with AnchoisFert afforded the best results. Also, the other vegetation parameter, the plant height, increased when going from plant grown in non‐fertilized soil to fertilized plants. The fruit parameter, however, dramatically changed. For plants grown in soil fertilized with AnchoisFert, the bulb weight increased by 65%, from 82 to 136 g, whereas the diameter rose by 133%, from 3 to 7 cm (**Figure** **1**).

Treatment with the other organic fertilizer, HM, increased the weight and diameter of the onion bulb in respect to control and similarly to NPK.

The analysis of cations and anions in red onion bulbs grown in pots for 3 months (maturation time) with the different fertilizers in the same amounts mentioned above revealed once again significant differences (**Table** **5**, results expressed in milligram per gram DW).

**Table 5:** Cations and anions detected in red onion bulbs grown in pots for 3 months (maturation time) with chemical (NPK), horse manure (HM), and AnchoisFert (AF) fertilizer

Cation	CTR [mg g^−1^]	NPK [mg g^−1^]	HM [mg g^−1^]	AF [mg g^−1^]
NH_4_^+^	8.7 ± 0.5^d)^	10.3 ± 0.7^c)^	31 ± 2^a^ ^)^	20 ± 3^b)^
K^+^	100 ± 4^a^ ^)^	96 ± 2^a^ ^)^	103 ± 3^a^ ^)^	102 ± 2^a^ ^)^
Mg^2+^	4.6 ± 0.9^b)^	4.9 ± 0.8^b)^	7.9 ± 1^a^ ^)^	7.5 ± 2^a^ ^)^
Ca^2+^	8.6 ± 1.2	10 ± 1.5^b)^	16 ± 2^a^ ^)^	15 ± 1^a^ ^)^
Anion	CTR [mg g^−1^]	NPK [mg g^−1^]	HM [mg g^−1^]	AF [mg g^−1^]
F^−^	0.47 ± 0.02^a^ ^)^	0.34 ± 0.04^b)^	0.16 ± 0.03^c)^	0.29 ± 0.02^b)^
Cl^−^	4 ± 0.5^b)^	16.4 ± 2^a^ ^)^	3.8 ± 0.9^b)^	19.4 ± 2^a^ ^)^
Br^−^	n.a.	0.8 ± 0.3^a^ ^)^	n.a.	0.7 ± 0.4^a^ ^)^
NO_3_^−^	1.2 ± 0.5^a^ ^)^	0.7 ± 0.2^a^ ^)^	1.1 ± 0.6^a^ ^)^	0.7 ± 0.3^a^ ^)^
NO_2_^−^	5.9 ± 1^a^ ^)^	4.7 ± 0.8^a^ ^)^	3.9 ± 0.9^a^ ^)^	3.7 ± 0.7^a^ ^)^
SO_4_^2−^	1.9 ± 0.3^b)^	35 ± 1.2^a^ ^)^	2 ± 0.3^b)^	41 ± 2^a^ ^)^
Malate	2.8 ± 0.6^a,b)^	3.6 ± 0.7^a^ ^)^	2.1 ± 0.1^b)^	2.4 ± 0.2^b)^

^a)^ Data expressed as mean of three replicates. Different letters indicate significant differences at *p* < 0.05.

Pointing to significant bioavailability of nitrogen present in horse manure chiefly as urea and ammonia, and in proteins in AnchoisFert, the amount of ammonium in red onion bulbs significantly increased, from 8.7 to 31 mg g^−1^ and to 20 mg g^−1^ when going from non‐fertilized soil to soils fertilized with HM and AF, respectively.

The amount of potassium slightly increased by 2% and 3% for onions growing in soils fertilized with the aforementioned organic fertilizers, but remarkably decreased by 4% for onions grown in soil fertilized with the NPK chemical fertilizer containing nearly pure potassium nitrate. Remarkably, the amounts of highly health‐beneficial Mg^2+^ and Ca^2+^ increased by 63% and 74% for onions grown in soil fertilized with the new marine fertilizer, and even more in the presence of horse manure (72% and 86%, respectively). The amount of Mg^2+^ remained nearly unvaried (from 4.6 to 4.9 mg g^−1^) for onions grown in the presence of the NPK chemical fertilizer, whereas the amount of Ca^2+^ increased by 16%.

The amount of toxic fluoride and nitrate ions decreased in all fertilized bulbs compared to control, noticeably more for fluoride in bulbs grown in soils fertilized with HM (from 0.47 to 0.16 mg g^−1^) and with AF (from 0.47 to 0.29 mg g^−1^). The decrease was more limited in the case of NPK fertilized bulbs (from 0.47 to 0.34 mg g^−1^). The amount of nitrate went from 1.2 to 0.7 mg g^−1^ for both AF and NPK fertilized bulbs. Pointing once again to large bioavailability of nitrogen in horse manure fertilizer, the decrease was negligible (from 1.2 to 1.1 mg g^−1^) for HM fertilized bulbs.

Chloride increased in NPK fertilized bulbs from 4 to 16.4 mg g^−1^, and even more for bulbs fertilized by the new marine fertilizer AnchoisFert when the concentration of chloride in bulbs reached 19.4 mg g^−1^ (a 4.85‐fold increase). Bromide was detected only in NPK (0.8 mg g^−1^) and AF (0.7 mg g^−1^) treated bulbs, likely due to the presence of bromide impurities in the NPK chemical fertilizer and of native sea bromide in fish biowaste. Nitrate and phosphate did not show significant differences between control and treatments. Sulfate, however, increased remarkably in NPK and AF treated bulbs, respectively going from 1.9 to 35 mg g^−1^ (a 18.4 increase) and from 1.9 to 41 mg g^−1^ (a 22‐fold increase). Malate slightly decreased in organically fertilized bulbs and increased from 2.8 to 3.6 mg g^−1^ in NPK treated bulbs (+28%).

Finally, results in **Table** **6** (expressed per unit weight of dried weight of red onion) show that fertilization barely affected the protein, carbohydrate, and fiber content in red onion bulbs grown in pots for 3 months (maturation time) with different fertilizers: NPK 20:10:10 (1.2 g/pot), horse manure (13 g/pot), and AnchoisFert (1.60 g/ pot).

**Table 6:** Total phenolic, flavonoid, protein and carbohydrate content, vitamin A, and fiber in red onion bulbs grown in pots for 3 months in non‐fertilized (CTR), chemically (NPK), horse manure (HM), and AnchoisFert (AF) fertilized soils

Parameter	CTR	NPK	HM	AF
Total phenols content [µg TAE/g]	14 040 ± 15^d)^	17 280 ± 33^c)^	18 930 ± 16^b)^	23 370 ± 27^a^ ^)^
Total flavonoid content [µg QE/g]	2601 ± 34^d)^	3305 ± 28^c)^	3866 ± 31^b)^	4914 ± 29^a)^
Vitamin A [µg g^−1^]	0.17 ± 0.02^c)^	0.39 ± 0.03^a^ ^)^	0.25 ± 0.01^b)^	0.26 ± 0.03^b)^
Total protein [mg g^−1^]	96 ± 2^b)^	105 ± 2^a^ ^)^	101 ± 4^a^ ^)^	97 ± 3^a^ ^)^

^a)^ Data expressed as mean ± standard error. Different letters indicate significant differences at *p* < 0.05.

The total phenolic content (TPC) and the TFC, however, significantly increased in fertilized red onion bulbs with respect to control, particularly in the case of organically fertilized onion bulbs. Once again, the greatest increases were observed in onions fertilized with AnchoisFert, followed by HM. In detail, the TPC went from 14 040 to 23 370 µg TAE/g, for AF treated bulbs, a 66% increase, whereas the TFC increased even more (by 89%) going from 2601 to 4914 µg QE/g. Fertilization with HM resulted in a 35% increase in TPC and a 48% in TFC. Fertilization with the NPK chemical fertilizer led to the lowest increase in TPC and TFC with respect to control, namely 23% and 27%. Only vitamin A (retinol being one of the main forms of vitamin A) reached the highest value (0.39 mg g^−1^) when soil was fertilized with the NPK chemical fertilizer.

## 3 Conclusions

In this contribution, we demonstrated how “AnchoisFert”, the solid residue comprised of milled anchovy leftovers after fish oil extraction with biobased limonene, is an exceptional organic fertilizer that was efficiently employed to promote the growth of Tropea's red onion (*A. cepa*), in less amount than horse manure (at about tenfold less). The fertilizer showed to be superior to commonly used organic (manure) and chemical (NPK) fertilizers.

Farmers looking for economically viable organic fertilizers capable to afford higher yields of valued horticulture crops find in AnchoisFert a soil and plant nutrient practically able to afford red onions of 65% higher bulb weight and 133% larger bulb diameter. Consumers in their turns would find on the marketplace Tropea's red onions, already known for their health benefits due to enhanced content of flavonols and anthocyanins,^[^ ^21^ ^]^ with 66% higher total phenolic and 75% higher TFC. The new organic fertilizer is rich in proteins, organic carbon, flavonoids, magnesium, potassium, phosphate, and sulfate can be used to promote the healthy growth of a variety of crops, replacing inorganic and organic fertilizers.

These findings close the anchovy fishing material cycle and dramatically improve the sustainability of both fishing and agriculture, thanks to the use of biobased limonene solvent derived from waste orange peel in a closed‐loop production cycle. The process indeed shifts fish oil and fish‐based fertilizer production from blue fish to blue fish waste,^[^ ^22^ ^]^ opening the route to the introduction of a new class of organic fertilizers of exceptional performance based on fish biowaste upgraded using a low‐cost, environmentally‐friendly circular process.

The newly extracted “AnchoisOil” fish oil will be commercialized in the omega‐3 marine lipids marketplace, whereas the dried fertilizer can be directly sold to farmers to be used in valued horticulture. In this way, the anchovy fillet production is completely converted into two highly valued bioproducts thanks to a zero‐waste circular production process in which the solvent, recovered after extraction of the AnchoisOil and the concomitant production of AnchoisFert, is made available for subsequent production cycles.^[^ ^8^ ^]^

Using this circular economy process no prolonged (several weeks long) composting of fish waste mediated by bacteria in composting plants is required to produce a nutrient‐rich fertilizer.^[^ ^23^ ^]^ Similarly, numerous fertilizers derived from fish‐waste are commercially available.^[^ ^24^ ^]^ Nearly all of them, however, are fish waste‐derived bioproducts requiring extensive further chemical, enzymatic, or thermal processing such as fish hydrolysate (requiring chemical, acid or alkali, or enzymatic hydrolysis followed by costly drying), fish emulsion (requiring heating and skimming process), or fish silage (a liquid product made from whole fish or parts of fish liquefied by natural enzymes). The treatment of fish biowaste used to extract the valued oil with antimicrobial limonene^[^ ^25^ ^]^ and producing at the same time a valued fertilizer, not only does not require any chemical or enzymatic treatment of fish waste, but also stabilizes the residual fish biowaste against microbial spoilage.

The results provide evidence that, at least in the case of Tropea's onion, a significantly lower amount of the new organic fertilizer AnchoisFert provides better results than commonly employed organic and inorganic fertilizers. In light of the forthcoming practical utilization of AnchoisFert as an organic fertilizer, it is also relevant that the use of AnchoiFert anchovy biowaste in place of manure will not increase the abundance (and the number) of antibiotic resistance genes in fertilized soil.^[^ ^26^ ^]^

Finally, the small amount of nicely scented fragrance and powerful antibacterial citrus limonene residual in AnchoisFert limits the formation of rotting fish odor typical of untreated fish biowaste due to trimethylamine rapid accumulation during storage.^[^ ^27^ ^]^ This study demonstrates the proof of concept directly on a valued horticulture (Tropea's red onion) generating revenues exceeding €25 million (for 25 000 tons production) in 2017.^[^ ^28^ ^]^ We are currently investigating the use of AnchoisFert in the fertilization of other valued horticulture crops.

## 4 Experimental Section

### Elemental Composition, pH, and Electrical Conductivity

Total and volatile solids and pH were evaluated according to standard methods.^[^ ^29^ ^]^ Carbon and nitrogen content (C/N) was measured using a total organic carbon analyzer TOC‐L_CSH_ (Shimadzu, Kyoto, Japan).

The analysis of the residual limonene in anchovy leftovers after fish oil extraction and solvent removal was carried out according to a previously reported procedure.^[^ ^30^ ^]^ A sample of the solid (0.3 g) was stirred with 3 mL of a toluene solution (as an internal standard) in cyclohexane (0.1 m) for 6 h, after which the suspension was filtered and a liquid aliquot injected into an offline GC‐FID Agilent 6890N gas chromatograph (Agilent, Santa Clara, CA, USA) equipped with a CP‐WAX 52CB column (60 m, i.d. 0.53 mm) also purchased from Agilent. The following GC‐FID method was applied: the injector was settled at 250 °C and the temperature program started from 50 °C (held for 5 min) up to 230 °C with a 10 °C min^−1^ rate (held for 10 min) and finally at 240 °C (held for 5 min) during the post run.

Electrical conductivity was determined using a HI5522 (Hanna Instruments, Woonsocket, RI, USA) conductivity meter. A 1:5 anchovy leftovers/water suspension (using distilled water) was mechanically shaken for 1 h at 15 rpm to dissolve the water‐soluble salts after which the authors measured the conductivity.

### Fertilization Experiment

In all experiments, the authors used a sandy‐loam (11.85% clay, 23.21% silt, and 64.94% sand) soil as defined in the World Reference Base.^[^ ^31^ ^]^ Each experiment was performed using pots of 30 cm diameter containing 9 kg of soil with a pH of 8.87 and 1.81% of organic matter. Pots were amended with AnchoisFert at the concentration of 1.60 g on the basis of its carbon content (40%). Non‐fertilized soil (CTR), organically fertilized soil with horse manure (Violmet Italy, Pisa), and chemically fertilized soil with NPK (Agricoltura Italia, Taranto) were used as controls. The experiments were performed in triplicates in greenhouse as previously reported.^[^ ^32^ ^]^

Pots were regularly watered to ensure that water content was maintained at 70% of field capacity. At the end of the experiments (90 days after treatments) the differently treated soils (three replicates) were air‐dried and sieved (<2 mm) prior to the chemical analysis. Soil samples for the biochemical determination (microbial biomass and enzyme activities) were stored in the refrigerator at 4 °C for up to 24 h until processing.

### Soil Analysis

Dry matter content of un‐amended and amended soils was determined at 105 °C until the mass loss of the sample during 24 h was lower than 0.5% of its weight. Electrical conductivity was measured with a HI5522 conductivity meter (Hanna Instruments, Woonsocket, RI, USA). Samples were suspended in distilled water (1:5 residue/water) and mechanically shaken at 15 rpm for 1 h to dissolve soluble salts. The pH was measured in distilled water (soil:water ratio 1:2.5) with a HI 2210 glass electrode (Hanna Instruments, Woonsocket, RI, USA).

Organic carbon and total nitrogen (TN) were assessed with conventional methods, namely the dichromate oxidation and Kjeldahl methods, respectively. Microbial biomass carbon was determined in field moist samples (equivalent to 20 g DW) by Vance's extraction method.^[^ ^33^ ^]^ Soil extracts of both fumigated and unfumigated samples were filtered and analyzed for soluble organic carbon. MBC was estimated on the basis of the differences between the organic C extracted from the fumigated soil and that from the unfumigated soil. An extraction efficiency coefficient of 0.38 was used to convert soluble C into biomass C.^[^ ^33^ ^]^

Water‐soluble phenols were extracted in triplicate as reported by Kaminsky and Muller.^[^ ^34^ ^]^ Total water‐soluble phenols (monomeric and polyphenols) were determined by using the Folin–Ciocalteu reagent. Tannic acid was used as a standard and the concentration of water‐soluble phenolic compounds expressed as microgram of tannic acid/gram of dry soil (µg TAE g^−1^ DS). Fluorescein diacetate hydrolase (FDA) was determined according to the method of Adam and Duncan.^[^ ^35^ ^]^ Dehydrogenase (DHA) activity was determined by the method of von Mersi and Schinner.^[^ ^36^ ^]^

Cations and anions were detected by ion chromatography using a Dionex ICS‐1100 ion chromotograph (Thermo Fisher Scientific, Waltham, MA, USA). For anion analysis, 0.5 g of dried material was stirred for 20 min using 50 mL of anion solution (Na_2_CO_3_/NaHCO_3_ 3.5 mm), filtering the extract through a Whatman 1 filter paper prior to the chromatographic analysis. For the cation analysis, 1 g of dry material was reduced to ash at 550 °C for 5–6 h in a porcelain capsule. The ash was then mineralized for 30 min at 100 °C using concentrated (1 m) HCl. The resulting solution was filtered through a Whatman 1 filter paper and analyzed by ion chromatography using 20 mm meta‐sulfonic acid as eluent.

### Plant Analysis

The fertilizers (fish waste, horse manure, NPK) were tested on a comparative basis on *A. cepa L*. (red onion). Each time, the experiment was terminated at bulb maturity as characterized by neck softening and reduced solution uptake. Bulb diameters were measured using a caliper. Leaf and root length were measured with a meter. Plants were harvested and separated into shoots, bulbs, and roots. After weighing the fresh plant and its parts, the latter were dried at 70 °C in an oven. Dry weights were determined and plant materials were ground to pass a 20‐mesh sieve. Antioxidant compounds and antioxidant activities in the differently fertilized onion bulbs were measured at the end of each growth cycle.

### Assessment of Phenolic Compounds in Bulb

Total phenol content was detected by the Folin‐Ciocalteu assay adapted to assess monomeric phenols and polyphenols red onion.^[^ ^37^ ^]^ The absorbance of each sample was recorded at 760 nm using a UV‐1800 high‐resolution spectrophotometer (Shimadzu, Kyoto, Japan). A calibration curve was constructed using gallic acid and results were expressed as micrograms of tannic acid per gram DW. Total flavonoids in the extracts were detected measuring the absorbance was measured at 430 nm. Flavonoid content was calculated from a calibration curve of quercetin and expressed as micrograms of quercetin per gram DW.

### Determination of Antioxidant Activities in Plants

The antioxidant activity against DPPH radical (2.2‐diphenyl‐1‐picryl‐hydrazyl‐hydrate) was determined according to a spectrophomometric method previously reported.^[^ ^38^ ^]^ The DPPH concentration in the cuvette was chosen to give absorbance values of ≈1.0. Changes in absorbance of the violet solution at 517 nm were recorded after 30 min incubation at 37 °C. The inhibition *I*(%) of radical‐scavenging activity was calculated as in Equation (1):

(1) $$\begin{array}{c} I\left(\%\right)=\left[\left(A_{0}-A_{S}\right)/A_{0}\right]\times 100 \end{array}$$

where *A*_0_ is the absorbance of the control and *A*_S_ is the absorbance of the sample following 30 min incubation.

The 2,2′‐azinobis(3‐ethylbenzothiazoline‐6‐sulfonic acid) diammonium salt (ABTS) radical cation decolorization assay was conducted measuring the absorbance at 734 nm according to a published method.^[^ ^39^ ^]^ The inhibition *I*(%) of radical‐scavenging activity was calculated from Equation (1) where *A*_0_ is the absorbance of the control and *A*_S_ is the absorbance of the sample after 4 min incubation. Results are expressed as µmol L^−1^ TE using a Trolox (1–50 µmol L^−1^) calibration curve. The oxygen radical absorbance capacity (ORAC‐fluorescein) assay was carried out according to a published method.^[^ ^40^ ^]^ An aliquot (20 µL) of the extract was added to a small sample (120 µL) of fresh fluorescein solution (117 nmol L^−1^). After an incubation time of 15 min at 37 °C, a sample (60 µL) of freshly prepared AAPH solution (40 mmol L^−1^) was added and fluorescence (λ_ex_ 485 nm, λ_em_ 520 nm) measured every 30 s for a 90 min overall analysis time. A blank sample using 20 µL of methanol was also analyzed. The ORAC values derived from a Trolox (10‐–00 µmol L^−1^) calibration curve are expressed as equivalent of Trolox micromoles per milligram fresh weight.

### Statistical Analysis

Analysis of variance was carried out for all the data sets. Significant difference tests were carried out to analyze the effects of fertilizers on each parameter measured. ANOVA and *t*‐test were carried out using the SPSS software for statistical analysis.^[^ ^41^ ^]^ Effects were considered significant at *p* ≤ 0.05.

## Conflict of Interest

The authors declare no conflict of interest.

## Author Contributions

A.M., R.C.: Conceptualization, Methodology, Supervision, Writing – Review and Editing; F.M., M.P.: Conceptualization, Writing‐ Original Draft, Funding Acquisition; M.T. and P.S.C.: Resource Acquisition, Methodology, Writing – Review and Editing; F.M.: Experimental Investigation, Methodology.

## Data Availability

The data that support the findings of this study are available from the corresponding author upon reasonable request.
